# What Is Our Current Understanding of PrP^Sc^-Associated Neurotoxicity and Its Molecular Underpinnings?

**DOI:** 10.3390/pathogens6040063

**Published:** 2017-12-01

**Authors:** Daniel Hughes, Mark Halliday

**Affiliations:** 1 MRC Toxicology Unit, Hodgkin Building, University of Leicester, Lancaster Road, Leicester LE1 9HN, UK; dh232@le.ac.uk; 2 Department of Clinical Neurosciences, University of Cambridge, Cambridge Biomedical Campus, Cambridge CB2 0AH, UK

**Keywords:** prion disease, neurodegeneration, neurotoxicity, proteostasis, PrP^Sc^

## Abstract

The prion diseases are a collection of fatal, transmissible neurodegenerative diseases that cause rapid onset dementia and ultimately death. Uniquely, the infectious agent is a misfolded form of the endogenous cellular prion protein, termed PrP^Sc^. Despite the identity of the molecular agent remaining the same, PrP^Sc^ can cause a range of diseases with hereditary, spontaneous or iatrogenic aetiologies. However, the link between PrP^Sc^ and toxicity is complex, with subclinical cases of prion disease discovered, and prion neurodegeneration without obvious PrP^Sc^ deposition. The toxic mechanisms by which PrP^Sc^ causes the extensive neuropathology are still poorly understood, although recent advances are beginning to unravel the molecular underpinnings, including oxidative stress, disruption of proteostasis and induction of the unfolded protein response. This review will discuss the diseases caused by PrP^Sc^ toxicity, the nature of the toxicity of PrP^Sc^, and our current understanding of the downstream toxic signaling events triggered by the presence of PrP^Sc^.

## 1. Introduction

The Prion diseases, or transmissible spongiform encephalopathies, are a group of fatal neurodegenerative diseases characterized by extensive neuronal death, spongiform change and gliosis. Uniquely among neurodegenerative disease, the prion diseases are transmissible, with infection between members of the same species, and in some cases between different species, possible. Since the discovery that the infectious component of prions is comprised solely of protein, more specifically a misfolded form of the cellular prion protein (PrP^C^), a great deal of research has focused on how this misfolded protein can cause such extensive and catastrophic damage to neurons. The major histopathological feature is the accumulation of extracellular amyloid plaques, comprised of misfolded PrP^C^ (termed PrP^Sc^ for PrP scrapie). However, it is not known how PrP^Sc^ causes neurodegeneration, or indeed what the exact toxic species is, as the link between PrP^Sc^, infectivity and toxicity is not sharply defined. The role of cellular PrP^C^ also remains elusive, further clouding the investigation into disease processes. Due to its insolubility, the structure of PrP^Sc^ has not been definitively proven, hampering rational drug discovery efforts, although recent advances have allowed various models to be proposed [1]. Unfortunately, and largely due to our poor understanding of these molecular mechanisms, therapeutic treatments for prion disease remain elusive.

Recent research has begun to unravel the role PrP^Sc^ plays in the neurodegeneration associated with prion disease. This review will discuss the diseases caused by PrP^Sc^ toxicity, the nature of the toxicity of PrP^Sc^, and our current understanding of the downstream toxic signaling events triggered by the presence of PrP^Sc^.

### 1.1. PrP^C^

The human prion protein is highly conserved in mammals, suggesting an essential role for the protein [2]. It is a small glycoprotein found mainly on the outer leaflet of the plasma membrane, held in place by a C-terminal glycosylphosphatidylinositol (GPI) anchor [3]. Although highly expressed in the tissues of the central nervous system (CNS), PrP^C^ is also expressed to varying degrees in most tissues in the body. Expression begins early in embryogenesis, and in adults the highest levels are in neurons, with moderate expression observed in glial cells and the peripheral nervous system [4]. Human PrP^C^ is synthesized as a 231 amino acid polypeptide (after removal of a 22 residue signal peptide [5]), which is processed through the endoplasmic reticulum (ER) and golgi apparatus. Post-translational modifications, including the removal of a signal sequence from the C-terminal end of PrP^C^, result in a mature protein of 208 amino acids in length. The main structural features are a globular C-terminal domain made up of three alpha helices with a small antiparallel beta sheet composed of two separate strands, and a largely unstructured flexible N-terminal tail [6].

The exact cellular function of PrP^C^ remains unclear, with several distinct and overlapping roles suggested. One of the most important proposed roles of PrP^C^ is the maintenance of myelination in the peripheral nervous system [7]. Interestingly, neuron-specific PrP^C^ expression is enough to maintain myelination, as although PrP^C^ is expressed in Schwann cells, it appears to not be essential there [7]. It is unknown what effect PrP^C^ has on CNS myelin. The first proposed role for PrP^C^ was in Cu^2+^ homeostasis due it its high affinity binding [8], although a functional physiological role for this affinity remains elusive. PrP^C^ has a putative role in protecting against stress, especially oxidative and some apoptotic stresses [9,10,11]. There is also evidence it helps to regulate neuronal excitability and memory [12,13]. Interestingly, it is also involved in the regulation of the circadian rhythm [14] and cellular differentiation [15,16]. The wide variety of biological roles has led to the suggestion that PrP^C^ is a scaffold protein that regulates the formation of a number of multi-protein complexes, but it is unlikely that the entirety of its physiological roles have been discovered [2].

### 1.2. PrP^Sc^

**Figure 1:**
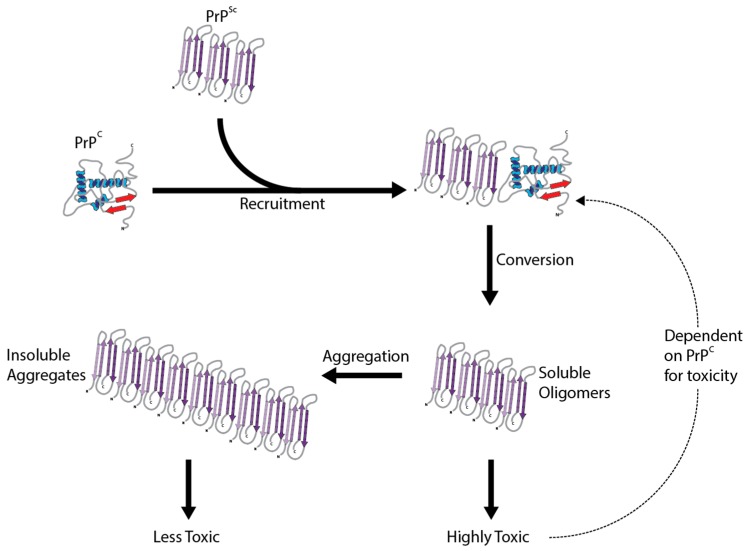
The conversion of PrP^C^ to PrP^Sc^. The protein only hypothesis of prion conversion posits that misfolded PrP^Sc^ acts a catalyst, directly binding to PrP^C^ and causing its conversion to PrP^Sc^. This self-perpetuating recruitment leads to large aggregates of PrP^Sc^, and underlies its infectious potential. Surprisingly, aggregated PrP^Sc^ appears to be minimally toxic, with smaller, soluble oligomers of PrP^Sc^ likely mediating the majority of the observed neurodegeneration. Importantly, PrP^C^ is required for both the conversion process and for the toxicity of PrP^Sc^ to manifest.

The crucial event in the development of prion disease is the structural and conformational change of PrP^C^ to the disease associated misfolded form, PrP^Sc^. This conversion changes PrP^C^ from a protein characterized by alpha-helices to a partially protease-resistant misfolded protein categorized by beta sheets [17]. Proteinase K (PK) partially digests PrP^Sc^ and is often used to determine the presence of misfolded PrP^Sc^ [18]. Despite this conversion being essential for the pathogenesis of prion disease, the molecular underpinnings are still not understood. The transformation is thought to be a post-translational change in conformation which initiates the catalytic conversion of PrP^C^ into more PrP^Sc^, by the interaction of existing PrP^Sc^ molecules (Figure 1). Continuous synthesis of PrP^C^ in the brain only provides more substrate for the pathological conversion to PrP^Sc^. While this mainly occurs after exposure to already misfolded PrP^Sc^, conversion can occur spontaneously in rare cases without exposure or a genetic basis. There are no primary sequence differences between PrP^C^ and PrP^Sc^, so the change is mediated by different secondary structures and a propensity to aggregate. This pathological change involving only the prion protein is summarized in the protein only hypothesis [19]. Strong evidence supporting PrP^Sc^ as being the main cause of prion disease comes from the production of infectious PrP^Sc^ in vitro [20]. Whatever the mechanism, this conversion is the basis for all the prion diseases.

### 1.3. The PRNP Gene

PrP^C^ is encoded by the *PRNP* gene found on chromosome 20 in humans, and chromosome 2 in mice. It is significantly conserved across mammalian species and even vertebrates as a whole. It contains three exons, but the entire open reading frame lies within exon 3 [21,22], with all of the disease-associated mutations discovered so far located within exon 3 [23]. The *PRNP* gene encodes a nonapeptide region followed by four octarepeats; this motif is thought to be important for its copper binding ability. More than 30 disease-causing mutations in *PRNP* have been discovered, leading to a single amino acid substitution, the addition of superfluous residues or an early truncation of the protein [24]. A number of insertion mutations have also been discovered in the octarepeat region. Many of these mutations are believed to facilitate the conversion of PrP^C^ to PrP^Sc^, linking these mutations to disease. There are also polymorphisms in the *PRNP* gene that can influence the risk of developing prion disease. The most important is at codon 129, as it predisposes to sporadic, iatrogenic and variant Creutzfeldt–Jakob Disease (CJD-see below) [25]. Codon 129 codes for either methionine (M) or valine (V), and M/M homozygosity predisposes to an earlier and more rapid onset of disease, while heterozygosity is protective. A glutamate to lysine substitution at codon 219 also appears to confer a protective effect against prion disease [26]. The shortest incubation times for prion disease occur when PrP^Sc^ and the host PrP^C^ share the same sequence, and when inoculation occurs intracerebrally instead of peripherally [27]. If the inoculating prion differs to the host PrP^C^, incubation times can be greatly increased, or clinical signs of disease never develop. This can prevent transmission between species, and is known as the species barrier.

### 1.4. Human Prion Disease

Human prion diseases are characterized by the presence of spongiform change, gliosis, amyloidosis and neuronal loss. Spongiosis appears as a series of vacuoles in fixed brain tissue. Astrocyte proliferation and neuronal cell death are other common features, and insoluble amyloid plaques containing aggregates of protease resistant prion protein (PrP^Sc^) are often correlated with prion diseases. Uniquely in the field of neurodegeneration, prion diseases are transmissible between members of the same species, and often between (mammalian) species, although not freely as species barriers do exist. They can be sporadic, familial or acquired in origin. The most common is CJD; others include Kuru, Fatal Familial Insomnia (FFI) and Gerstmann–Straussler–Scheinker (GSS) disease. Although all are caused by the misfolding of PrP^C^, these diseases often display startlingly different pathological and biochemical characteristics. These diseases can also affect different regions of the brain, causing further differences in disease course and symptoms.

Mutations in *PRNP* cause inherited prion disease that accounts for approximately 15% of prion disease cases, producing a wide spectrum of clinical phenotypes [28]. Inherited prion diseases generally have an earlier onset, but slower disease progression than sporadic cases. These mutations are autosomal dominant, and can result in either an expanded octapeptide repeat in the normal sequence of the prion protein, a non-conservative point mutation or a stop codon insertion in the *PRNP* open reading frame (ORF). This can lead to familial CJD (fCJD), GSS and FFI. fCJD causes a rapidly progressive dementia with myoclonus and abnormal electroencephalogram (EEG) recordings, GSS is characterized by a slow progression of ataxia and late onset dementia, and FFI is unique with its refractory insomnia, dysautonomia and motor dysfunction. These disease syndromes are not absolute; however, the same mutation can lead to highly divergent phenotypic and pathological variation between individuals [29].

Sporadic CJD (sCJD) accounts for 85% of cases of human prion disease, occurring in around one in a million people over the age of 65. Early onset cases are extremely rare. The disease presents with a rapidly progressive dementia with myoclonus and development of movement disorders such as tremor and rigidity. Associated neurological symptoms include cerebellar ataxia, pyramidal and extra pyramidal signs, and cortical blindness. Most cases have a characteristic EEG that includes periodic sharp-wave complexes. Death occurs after an average of 4 months from diagnosis, making it one the most aggressive forms of neurodegeneration [30].

Acquired prion diseases include Kuru, iatrogenic CJD (iCJD) and vCJD. Kuru is caused by the eating of infected brain tissue, and is characterized by progressive cerebellar ataxia, mood and personality changes, and a late onset dementia [31]. Death occurs approximately one year after the emergence of clinical symptoms. iCJD is rare, and has occurred after the exposure of patients to contaminated medical treatments or equipment. Contaminated dura matter and corneal grafts, inoculation with human pituitary-derived growth hormone and gonadotrophins have all been reported [32]. Improperly sterilized surgical equipment has also led to iCJD after brain surgery. iCJD caused by intracerebral infection is relatively rapid in onset and duration, with prominent early dementia. In contrast, peripheral inoculation is associated with a prolonged incubation time and late onset dementia.

In the mid-1990s, in the wake of the Bovine Spongiform Encephalopathy (BSE) epidemic, a new neurodegenerative illness emerged in the UK. Clinically and pathologically, it resembled sCJD, but the disease had a longer duration with a protracted neuropsychiatric syndrome, and critically, mainly affected young people [33,34]. After the realization, it was a new disease, it was termed new variant, or variant CJD (vCJD) [35]. The age of onset was much earlier than sporadic CJD, with a mean age of 29, and patients as young as 16. The initial symptoms are mainly behavioural, followed by ataxia and movement disorders. Dementia occurs at a much later point in the disease than CJD, with EEG abnormalities frequently absent. It also progresses slower than sporadic CJD, with a mean duration of 14 months. As none of the patients had *PRNP* mutations and were at a very low risk of iatrogenic exposure, BSE was considered to be the most likely cause. Molecular studies on vCJD tissue showed that the biochemical properties of the protease-resistant prion protein found in these patients were distinct from other human prion diseases [36], but similar to that of BSE [37], leading to the acceptance that BSE exposure causes vCJD.

### 1.5. Animal Prion Diseases

**Figure 2:**
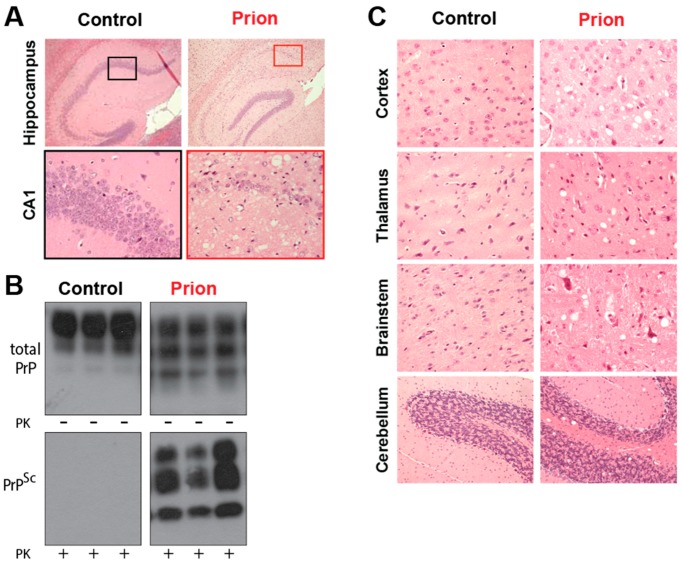
The neuropathology of prion disease. (**A**). The Rocky Mountain Laboratory (RML) strain of prion disease induces extensive neurodegeneration in mice, especially in the hippocampus where considerable neuronal death is observed in the CA1 region (hematoxylin and eosin stained hippocampal sections from uninfected control mice, or prion-infected terminal tg37^+/−^ mice) (**B**). Prion infection is associated with the accumulation of PrP^Sc^, which is often detected by its partial proteinase resistance to digestion by proteinase K (total PrP and PrP^Sc^ levels, detected with and without proteinase K digestion) (**C**). Spongiosis, an intracellular oedema that appears as holes in histological slices after fixation, is observed throughout the brain in both human and animal cases of prion disease (hematoxylin and eosin stained hippocampal sections from uninfected control mice, or prion-infected terminal tg37^+/−^ mice).

Several mammalian species are also afflicted by prion disease, including scrapie in sheep, Chronic Wasting Disease (CWD), which mainly affects deer and elk in North America and transmissible mink encephalopathy, which affects mink feeding on infected livestock. Unlike in humans, most cases are infectious in origin, although the increased surveillance for prion disease after the BSE outbreak is identifying increasing numbers of spontaneous cases. The histopathological features are grossly similar between human and animal prion disease. BSE affects the brainstem of cattle causing ataxia, with a presymptomatic incubation time of 5 years [38]. CWD is thought to be highly contagious due to the high number of animals infected despite the free-ranging habits of deer and elk. It has spread through much of North America and been detected in South Korea [39,40]. CWD has recently been observed in free ranging reindeer in Norway, with the origin of this outbreak currently unknown [41]. Despite not being natural carriers of prion disease, mice have been used extensively to model prion disease. They have been infected with sheep scrapie, and genetic modification of the carried *Prnp* gene allows prion disease from other hosts to be replicated (Figure 2).

### 1.6. Selective Neuronal Vulnerability in Prion Diseases

The same disease agent, PrP^Sc^, is associated with all the prion diseases, however, the signs and symptoms of each disease can differ dramatically. This may be due to the regional differences of PrP^Sc^ accumulation in the brain and the neurons affected. This is thought to be a result of many factors including specific interactions of different protein conformers as well as region-specific micro-environments which contain a different combination of metals, chaperone proteins and translational machinery [42].

For instance, in FFI, neurodegeneration occurs in the thalamus, accounting for the insomnia associated with this prion disease due to the involvement of the thalamus in sleep regulation [30,43]. In Kuru, the damage often occurs in the cerebellum, leading to defects in coordination, while GSS has a wider range of clinical manifestations ranging from cerebellar ataxia to spastic parapesis, often in combination with dementia [43]. In CJD, the cerebral cortex is the main affected brain region [44], which results in mental impairments, mood change and various visual disturbances.

## 2. Is the Prion Protein Directly Responsible for Prion Disease?

Despite initial resistance, the protein only hypothesis of prion disease is now widely accepted. However, many unanswered questions remain. The most pressing, and to this day still the most elusive, is how exactly does the conversion of PrP^C^ to PrP^Sc^ cause prion disease? There are several possibilities that have been suggested to be a cause: the conversion of PrP^C^ to PrP^Sc^ causes a toxic loss of function in the PrP^C^ protein; PrP^Sc^, or aggregates of PrP^Sc^, are directly toxic to neurons; the conversion process itself is somehow toxic, or there are transient intermediaries formed that mediate the toxicity. In addition to the main cause, multiple toxic downstream processes are likely to be engaged, with neuroprotective responses failing or behaving ineffectively.

### 2.1. PrP^C^ is Essential for Prion Disease

It is now known that PrP^C^ loss of function is not the main cause of prion disease. Knockout mice models are grossly normal and display no obvious phenotypes [12,45,46]. In species where prion disease is naturally occurring, such as cows and goats, PrP knockout is again non-toxic [47,48]. Therefore, loss of PrP^C^ function does not cause prion-induced neurodegeneration. However, since the first reports of PrP^C^ knockout, many subtle phenotypes have been described, including some related to neuroprotective pathways such as neurogenesis and stress protection [49]. Although not a direct cause, impairment of some of these processes might contribute to disease under specific stress insults.

PrP^C^ does appear to have some directly neuroprotective abilities. PrP^C^ is upregulated in neurons after ischaemic stroke in humans, and knocking out PrP^C^ was shown to greatly increase infarct size in an animal model [50]. PrP^C^ is involved in the maintenance of myelin in the mouse peripheral nervous system [7]. PrP^C^ is also involved in protecting against the neurotoxicity induced by the artificial expression of its closest homologue, doppel (Dpl). This was first discovered due to the accidental expression in the brain of Dpl by a group trying to delete the Prnp gene in mice [51], caused by the fusion of the PrP promoter to the Dpl open reading frame and its subsequent ectopic expression in the brain [52]. Dpl expression caused Purkinje cell death [51] and a late onset ataxia [53] that could be rescued by PrP^C^ expression in a dose-dependent manner [54]. However, Dpl is not normally expressed in the CNS in any significant quantities, and levels are not increased during prion disease [55], so the physiological extent of neuroprotection by PrP^C^ cannot be inferred.

The most important observation from PrP^C^ knock out experiments is the absolute and total requirement of the presence of PrP^C^ for any PrP^Sc^ induced pathology. This was first demonstrated in mice lacking PrP^C^, which were resistant to prion infection [56]. A set of elegant experiments further proved this effect. Brandner and colleagues grafted neural tissue overexpressing PrP^C^ into the brain of PrP null mice [57]. After inoculation with prions, the grafts accumulated high levels of PrP^Sc^ and developed the severe histopathological changes characteristic of prion disease. Substantial amounts of graft-derived PrP^Sc^ migrated into the surrounding areas of the host brain, but even 16 months after inoculation no pathological changes were seen in PrP null tissue [57]. Therefore, in addition to being resistant to scrapie infection, brain tissue devoid of PrP^C^ is not damaged by exogenous PrP^Sc^, providing further evidence that PrP^Sc^ is not directly toxic in vivo. This was further demonstrated in experiments in which PrP^C^ was depleted during the course of prion infection [58]. Double transgenic mice were generated that had floxed PrP transgenes, from which the PrP coding sequence is deleted by neuronal Cre expression at 9 weeks of age. When these mice are inoculated with prions before PrP knockout, they develop the initial stages of prion disease, including spongiosis and hippocampal shrinkage. When the Cre-mediated excision of the Prnp gene occurred, prion disease was prevented from developing and the early spongiform changes were reversed, despite continued prion replication in non-neuronal cells and further astrocytic extra-neuronal PrP^Sc^ deposition. The mice lived for the normal lifespan, and remarkably, they never developed further clinical disease [58]. These results also argue against direct neurotoxicity of PrP^Sc^, because the continued non-neuronal replication and accumulation of PrP^Sc^ throughout the brains of scrapie-infected mice was not pathogenic.

### 2.2. The Weak Links between PrP^Sc^ and Neurotoxicity

A number of other experiments have also demonstrated the complicated relationship between PrP^Sc^ and toxicity. Interestingly, the GPI-anchor has been demonstrated to be required for PrP^Sc^ induced toxicity. Prion inoculated mice expressing anchor-less PrP which is released into the extracellular space instead of being tethered to the plasma membrane, freely replicate PrP^Sc^ but do not develop disease [59]. This again suggests that PrP^Sc^ is not directly toxic to neurons, and also that either conversion at the membrane or subsequent internalization mediated by the GPI anchor is required for toxicity. In concert with this, the levels of PrP^Sc^ deposition are poorly correlated with disease progression, with subclinical cases of prion infection and prion disease observed with low prion titers observed in both animal and human cases [60]. Several studies have noted neurodegeneration without amyloidgenic PrP^Sc^ when passaging BSE prions into mice or rats [61,62]. In humans, several inherited mutations cause neurodegeneration without plaques [63,64,65]. A transmembrane form of the prion protein has been demonstrated to cause GSS without any detectable PrP^Sc^ [66]. Studies using hamster prions injection into mice have demonstrated cases of substantial PrP^Sc^ replication without the emergence of clinical signs [67,68]. These experiments have profound implications for the development, diagnosis and treatment of prion disease. It may be that PrP^Sc^ is a better marker for prion infection rather than prion-induced neurodegeneration, and again demonstrates the relative lack of neurotoxicity of PrP^Sc^. Subclinical infection also poses a public health risk, as PrP^Sc^ from non-symptomatic individuals can still be infectious to other, perhaps more susceptible individuals [69].

Unfortunately, the identity of the actual infective agent even in purified scrapie infectious fractions remains a source for debate. Only 1 in 10^5^ particles appears to be infectious [70], so the structure of the infectious agent cannot be definitively inferred. The most infectious particles have been shown to be non-fibrillar in nature, and comprised of 14–28 PrP molecules, with infectivity significantly reduced in oligomers larger or smaller than this [71]. PrP^Sc^ is partially protease resistant by definition, but infectious PK-sensitive forms of PrP^Sc^ have also been detected [72,73,74]. These PK-sensitive forms share structural features with PK-resistant PrP^Sc^, and are similarly infective [74]. In contrast, high levels of PK-resistant PrP^Sc^ are not always correlated with disease [75], or infectivity [76]. Partial protease resistance is not consistently correlated with infectivity, and non-infective protease resistant PrP can be produced [77]. Furthermore, infectious PrP^Sc^ can be comprised of both protease sensitive and resistant fractions. All of these studies highlight the complexity and heterogeneity of the toxic agent in prion disease, obfuscating a better understanding of the mechanisms of disease.

### 2.3. PrP^Sc^ Structure and Toxicity

A puzzling characteristic of prion disease is the existence of different strains/isolates of PrP^Sc^ that when inoculated into model organisms can produce drastically different incubation times, clinical signs and pathology. Biochemically, the strains display different immunohistopathological characteristics and protease sensitivities [78]. As prions are comprised only of protein and are generated by the conversion of host PrP^C^, the prion strain phenomenon cannot be attributed to genetic variability. Instead, prion strains are likely to result from distinct conformational changes, that are maintained during the conversion process [79]. This suggests that the structure of PrP^Sc^ may help to explain the associated neurotoxicity. Unfortunately, difficulties in purifying and determining the structure of PrP^Sc^ have hindered investigations.

The toxic conversion results in PrP^Sc^ characterized by extensive β-sheet secondary structure [17], a protease resistant core [80] and new epitopes not shared with PrP^C^ [81]. From here, monomers, oligomers, protofibrils and insoluble fibrils of PrP^Sc^ then accumulate, creating a heterogeneous assortment of structures. It is believed the β-sheet is essential for the aggregation of PrP^Sc^ into amyloid fibrils [82]. Several models of PrP^Sc^ amyloid have been suggested, including short compact fibrils or parallel β-sheets (see [1] for review). It is believed that fibrils dictate infectivity and the species barrier effect [1], as fibril load correlates with infectivity, but not toxicity [83]. Even if they are not the toxic species, their presence may catalyze the formation of a more toxic species [84], with growing evidence pointing towards oligomers of PrP^Sc^ as being the culprit. They display increased toxicity compared to fibrils both in vitro and in vivo [85,86,87]. These results are in agreement with the current evidence from other protein misfolding neurodegenerative diseases, where smaller oligomer molecules are thought to be the most toxic [88]. Determining the exact structure of the various PrP^Sc^ species remains elusive, and thus so does the exact relationship between PrP^Sc^ structure and toxicity. There is evidence that the disordered N-terminal domain of PrP^C^ mediates the toxicity of PrP^Sc^ [89]; it is likely that a better understanding of similar relationships between structure and function will improve our understanding of PrP^Sc^ neurotoxicity.

## 3. The Molecular Underpinnings of PrP^Sc^ Toxicity

Despite the weak evidence for the direct neurotoxicity of PrP^Sc^, there are numerous reported detrimental effects of PrP^Sc^ formation/aggregation that explain at least some of the toxicity of prion disease. This includes the induction or inhibition of a range of cellular processes, discussed below.

### 3.1. Autophagy

Autophagy is the cell’s main clearance mechanism for aggregated or dysfunctional proteins, which delivers cytoplasmic macromolecules or organelles to be degraded to the lysosomes. The structure to be degraded is enclosed in a double membrane structure termed the autophagic vacuole (or autophagosome), which then fuses with a lysosome containing hydrolases and digestive enzymes. Autophagic processes can ultimately initiate a form of cell death similar to apoptosis if levels of aggregated protein are deemed to be too high. Due to the large buildup of aggregated proteins in the protein misfolding neurodegenerative diseases, it is no surprise that dysfunction of this pathway is often observed, and increasingly autophagy is being explored as a possible therapeutic target. The links between autophagy and prion disease were first described in a hamster model of prion disease [90], where large autophagy vacuoles were observed, which increased in size as the disease progressed. Since then, autophagic vacuoles have been observed in many experimental models and human patients [91,92,93,94].

Pharmacological treatments that induce autophagy have conferred neuroprotection in various models of prion disease. Lithium, astemizole and the experimental compound FK506 have all been reported to induce autophagy and prolong survival in prion infected mice, with the authors attributing the neuroprotective effects to an increased removal and degradation of PrP^Sc^ [95,96,97]. The mTOR inhibitor rapamycin, which also induces autophagy, was shown to be neuroprotective in a mouse model of GSS [98]. Despite these results, it is still unclear if reduced or dysfunctional autophagy is a direct toxic effect of PrP^Sc^, or if inducing autophagy confers neuroprotection by increasing clearance of PrP^Sc^ preventing other toxic downstream effects from occurring.

### 3.2. The Induction of Apoptosis

Apoptosis is the programmed death of cells, and is characterized by cell shrinkage, condensation of the chromatin and fragmentation of the nucleus. It is an active process requiring gene transcription and protein translation, and markedly different from the uncontrolled necrosis [99]. Apoptosis occurs in both experimental prion disease and human patients [100,101,102,103,104]. However, it is likely that the apoptosis observed in prion disease is a downstream effect of the prion infection, rather than the direct cause of PrP^Sc^ toxicity. Genetic deletion of the major pro-apoptotic protein Bax has no effect on prion disease progression [105], and over expression of the anti-apoptotic Bcl-2 again has no effect [106]. Deletion of caspase-12, a pro-apoptotic protein induced by ER stress, also failed to protect neurons in prion diseased mice [107].

### 3.3. Proteasome/Ubiquitin Inhibition

The Ubiquitin Proteasome (UPS) system is the main route for targeted protein degradation in mammalian cells. Degradation of proteins by the UPS occurs over two stages; firstly, the protein to be degraded is conjugated with multiple ubiquitin (Ub) molecules, targeting it for destruction. The Ub tagged protein is then degraded by the 26S proteasome [108] and broken down into its constituent peptides, which can be recycled for future protein synthesis.

Numerous studies suggest that disruption of the UPS by PrP^Sc^ in prion disease is a contributing factor to pathogenesis. An increase in ubiquitin immuno-reactivity, which is indicative of UPS dysregulation, has been reported in prion-infected mice in the early stages of disease and precedes behavioural deficits as well as correlating with PrP^Sc^ deposition [109].

**Figure 3:**
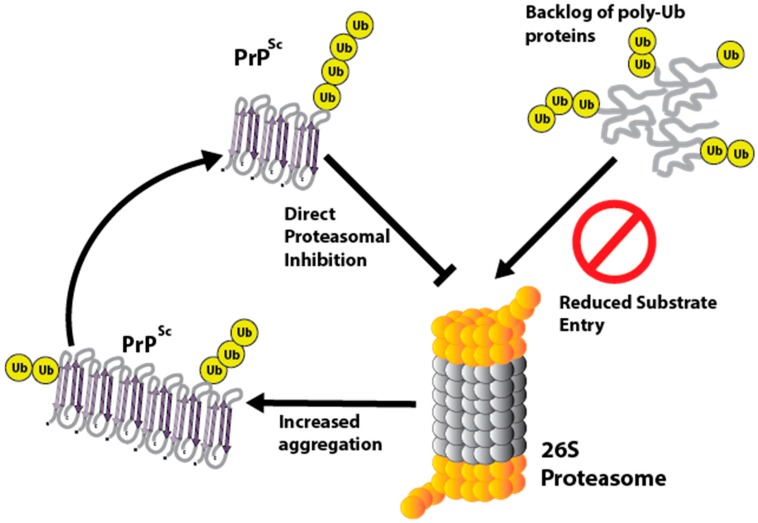
Disruption of the ubiquitin proteasome system by PrP^Sc^. PrP^Sc^ can directly inhibit the 26S proteasome by binding to the 20S subunit, preventing substrate entry. The causes increased PrP^Sc^ aggregation and accumulation of poly-ubiquitinated proteins in the cytoplasm.

PrP isoforms have been shown to directly inhibit the 26S proteasome [109,110,111,112,113]. In vitro data using purified proteasomes and three different cell lines show that PrP oligomers directly inhibit the 26S proteasome [112]. It is thought that PrP molecules rich in β-sheet conformations such as PrP^Sc^ inhibit the 26S proteasome by reducing gate opening of the 20S subunit and hence limiting substrate entry [111]. This has been inferred because β-sheet rich PrP does not inhibit mutant proteasomes with a constitutively open gate [110]. Inhibition of the proteasome can also increase aggregation of PrP^Sc^, in addition to affecting the processing of PrP^C^ [114,115]. This could result in a positive feedback mechanism where PrP^Sc^ induces proteasomal malfunction, which in turn leads to increased aggregation of PrP^Sc^ and a backlog of poly-ubiquitinated proteins (Figure 3).

Proteasomal dysfunction is a common feature of many neurodegenerative diseases and given that PrP^Sc^ can directly inhibit the UPS, this could make activation of the UPS a possible therapeutic target in prion disease. McKinnon et al., found that activation of the UPS by a small molecule inhibitor resulted in enhanced clearance of poly-ubiquitinated substrates and reduced PrP^Sc^ load in prion infected cells [109]. Reducing PrP^Sc^ and its effects on proteostasis in this manner could be doubly beneficial.

### 3.4. The Unfolded Protein Response

Recent studies have implicated aberrant signaling by the unfolded protein response (UPR) as a major pathological player in prion disease progression and neuronal cell death [116,117,118,119,120,121,122]. The UPR is made up of three signaling cascades all beginning in the endoplasmic reticulum membrane. They are controlled by PKR-like endoplasmic reticulum kinase (PERK), Inositol-requiring enzyme 1 (IRE1) and Activating transcription factor 6 (ATF6) [123,124,125], and all are activated in response to an unfolded protein load within the ER. PERK phosphorylation attenuates protein translation via the phosphorylation of eIF2α at serine 51, in an attempt to prevent additional unfolded protein production, and a number of stress response genes are upregulated by ATF6, XBP1 (a transcription factor downstream of IRE1) and ATF4 (a transcription factor induced by eIF2α phosphorylation) [126]. After the resolution of unfolded protein stress, GADD34, the eIF2α-targeting component of protein phosphatase 1, reduces eIF2α phosphorylation and restores translation [127].

A series of studies using Tg37 hemizygous mice infected with Rocky Mountain Laboratory (RML) prions identified aberrant UPR signaling as a pathogenic mechanism in prion disease. These mice overexpress wild type murine PrP around 3-fold normal levels and follow a well-documented disease progression after prion inoculation. Loss of synapse regeneration is evident at 7 weeks post inoculation (wpi), and PERK activation and phosphorylation of eIF2α at 9 wpi precedes neuronal loss, which is apparent at 10 wpi [122]. Lentivirally expressing GADD34 reduces eIF2α phosphorylation, restores translation and prevents neurodegeneration. In contrast, administration of salubrinal (an inhibitor of eIF2α dephosphorylation) accelerates disease in these mice. Interestingly, targeting the PERK pathway in this model confers neuroprotection without affecting levels of PrP^C^ or PrP^Sc^. In a subsequent study, prion-infected Tg37 hemizygous mice were treated with GSK2606414 [121] a potent and bioavailable PERK inhibitor [128]. This compound effectively prevented neurodegeneration, even when dosed after the emergence of early neurological disease indicators. Again, this protection was independent of PrP^Sc^ levels.

Another compound, ISRIB (integrated stress response inhibitor), which acts downstream of eIF2α phosphorylation, has been used to successfully delay neurodegeneration in prion-infected mice [119]. ISRIB binds to eIF2B, a guanine exchange factor essential for supplying the energy needed for the initiation of translation, stabilizing it in its dimeric form [129]. This allows eIF2B to act as a guanine exchange factor even in the presence of eIF2α-P, which normally inhibits this action. The result is a partial restoration of protein synthesis in ER-stressed conditions. Administration of ISRIB to prion mice resulted in an increase in survival and marked neuroprotection of hippocampal neurons, with the treated mice also performing better in behavioural tests [119]. These studies validate PERK and the PERK-eIF2α pathway as therapeutic targets, with the restoration of translation proposed to be the neuroprotective process.

A further study aimed at repurposing drugs for use in neurodegeneration identified two molecules, trazodone and dibenzoylmethane (DBM), that partially restore protein synthesis rates during ER stress [120]. Both were neuroprotective in prion disease and a model of fronto-temporal dementia [120]. Trazodone is a serotonin agonist and reuptake inhibitor that can be rapidly repurposed, and DBM is a curcumin analogue under investigation as an anti-cancer agent.

**Figure 4:**
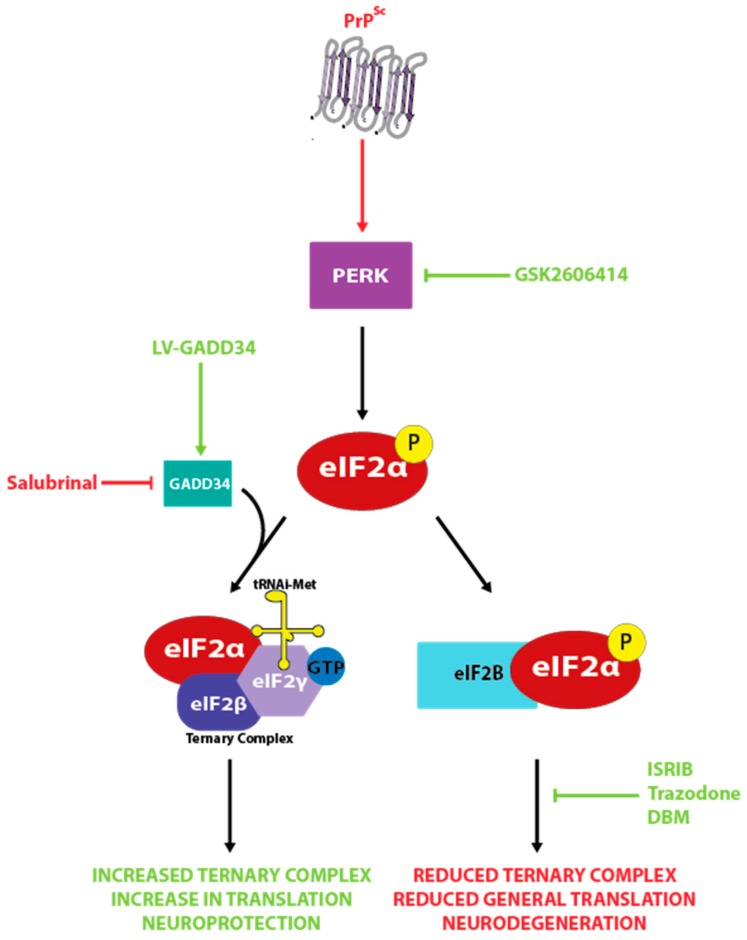
The role of the unfolded protein response (UPR) in prion neurodegeneration. Aggregates of PrP^Sc^ activate PERK signaling, leading to a reduction in protein synthesis rates mediated by the phosphorylation of eIF2α. This starves neurons of essential proteins, leading to neurodegeneration. Restoring translation rates via lentiviral expression of GADD34 or treatment with a variety of small molecule inhibitors increases translation and is substantially neuroprotective.

The results of these studies suggest that the decline in protein synthesis rates mediated by PERK pathway signaling is a main contributing factor to prion disease associated neurodegeneration (Figure 4). Neuroprotection was observed despite the continued replication of PrP^Sc^, further demonstrating the lack of direct toxicity of PrP^Sc^ and instead implicating downstream processes as mediating the toxicity in prion disease.

### 3.5. Oxidative Stress

Increased oxidative stress and prion disease have been linked in a plethora of previous studies. However, it is unclear if prion disease progression causes increased oxidative stress or if prion-associated pathology results from oxidative stress.

Conversion of PrP^C^ to PrP^Sc^ as an effect of oxidative stress has been reported in various in vitro studies [130,131,132]. It has been postulated that prion misfolding in disease, particularly those of a sporadic nature, could be triggered by oxidative stress. It has also been suggested that PrP^C^ plays a role in cellular resilience to oxidative stress via copper metabolism associated with PrP^C^ and superoxide dismutase (SOD) activity [133,134]. Loss of the antioxidant functions of PrP^C^ after conversion to PrP^Sc^ could hence contribute to pathogenesis of prion diseases. Studies performed in scrapie-infected mice and hamsters show an increase in markers of oxidative stress such as heme oxidase 1 [135] during disease progression. Alterations in free radical metabolism and increased oxidative stress can cause mitochondrial dysfunction in the brains of scrapie-infected animals, suggesting that this mitochondrial dysfunction is a contributing factor to prion disease progression [136,137].

### 3.6. Synaptic Dysfunction

One of the starkest phenotypes of prion disease is the gradual but continuous loss of synapses as the disease progresses. In mice, the number of synapses progressively decreases across a number of brain regions, including the hippocampus, with both pre- and post-synaptic processes affected [138,139,140,141,142]. This loss of synapses is correlated with a number of behavioural phenotypes [122,143,144], and precedes neuronal loss, suggesting that it is the loss of synapses that is causing the behavioral phenotypes. In human disease, there is also evidence for large quantities of synaptic loss and neurological deficits without extensive neuronal loss [145], but examples are limited due to the necessity of waiting until a post-mortem for investigation, where neuronal loss leaves a much more catastrophic signature than synaptic dysfunction.

PrP can modulate the activity of N-methyl-d-aspartate (NMDA) receptors, an important ion channel involved in long-term potentiation, but also to the detriment of neurons during excitotoxicity. In prion disease, an increase in activity through NMDA receptors is observed facilitating neuronal death, which can be attenuated by expressing PrP^C^ [146,147,148]. There is also evidence for PrP^Sc^ directly forming pores in the plasma membrane, disrupting the electrochemical balance of the neuron [149]. Here, soluble oligomers of misfolded PrP insert into the plasma membrane forming ion channel like structures that allow the passage of ions, causing homeostatic disruption [150].

### 3.7. Microglia

Microglial accumulation is observed in a range of neurodegenerative diseases, including prion disease. Microglia are the macrophages of the CNS and release cytokines in response to a wide variety of stressors. There is some evidence that microglia can actually be involved in the dissemination of PrP^Sc^ throughout the brain. Baker and colleagues demonstrated that purified microglia from CJD-infected mice show similar infectivity to crude homogenate, despite having 50x less PrP^Sc^ [151]. Microglia have been shown to be required for the toxicity of a human PrP fragment in vitro, while release of reactive oxygen species (ROS) from activated microglia is suggested as the cause of neuronal apoptosis [152,153].

However, one study [154] suggests a protective role of Cx3cl1/Cx3cr1 signaling in prion disease. The chemokine Cx3cl1 is expressed by neurons and its receptor Cxcr1 is solely expressed by microglia. Prion incubation times in Cx3cr1 null mice were significantly reduced, with no observed changes in microglial activation or chemokine/cytokine expression.

Brain slices with microglia ablation aggravated prion-induced neurotoxicity, and IL34^−/−^ mice (which present with reduced microglia) amplified deposition and accelerated prion disease [155]. However, contrasting work suggested microglia are not efficient at clearing PrP^Sc^ [156].

## 4. Discussion

The prion diseases display a unique pathological and biochemical profile characterized by spongiosis, astrocytosis and neuronal death. These disorders are accompanied by a unique set of biological features, and remain among some of the most puzzling and inscrutable diseases known. Uniquely for a transmissible disease, the infectious agent is a protein encoded by the host’s own genome. The protein only hypothesis of prion disease is now widely accepted, but many unanswered questions still remain. At the center of the prion phenomenon is the conversion of PrP^C^ to PrP^Sc^, usually followed by the widespread aggregation of PrP^Sc^ throughout the brain. One of the first major milestones of prion disease research was the discovery that disease was not caused by the loss of function of PrP^C^ [56]. Subsequent research has uncovered a number of proposed cellular roles for PrP^C^, including some neuroprotective processes [2], so the loss of function of PrP^C^ cannot be completely ruled out as a contributor to pathology, especially as levels drop during the disease course [157]. Another interesting finding, and one that has yet to be fully explored, is that PrP^C^ is required for the toxicity of prion disease to manifest [57,58]. Uncovering why will surely be a major step in the search for a cure for prion disease.

If the loss of function of PrP^C^ is not the main pathological cause, then the aggregation of PrP^Sc^ becomes the most likely suspect for the mediator of toxicity. Unfortunately, prion disease is rarely so simple. Aggregates of PrP^Sc^ correlate poorly with disease progression, subclinical cases of prion disease with large amounts of PrP^Sc^ have been discovered, and prion disease without aggregates of PrP^Sc^ has also been observed [68,69]. In addition, the extra-neuronal replication of PrP^Sc^ by glial cells is not toxic to neurons if they no longer express PrP^C^ [58]. Even the infectivity of PrP^Sc^ is not fully understood, as only a tiny proportion of PrP^Sc^ appears to be infectious, and protease resistance, usually the main marker of the presence of PrP^Sc^, is poorly correlated with infectivity [71,75,76].

At the heart of prion disease is the structural change from α-helical PrP^C^ to β-sheet rich PrP^Sc^ [17]. This, along with the puzzling prion strain phenomenon, suggests that determining the structure of PrP^Sc^ and its various misfolded states will greatly illuminate the molecular mechanisms of PrP^Sc^ associated toxicity. Unfortunately, this has proven extremely difficult due to the insolubility of PrP^Sc^, hampering our understanding. However, growing evidence is suggesting that it is oligomers of PrP^Sc^ that are most toxic, compared to the larger fibrils of PrP^Sc^ or single monomers [158]. Why PrP^C^ is more prone to misfolding than other proteins, and exactly how this happens, especially in sporadic cases where no template of PrP^Sc^ is present, is an extremely important topic of research. It has been suggested that there are cofactors that may contribute to the misfolding [159], but so far no definitive explanation has been found. Another suggestion is that an intermediate between PrP^C^ and PrP^Sc^ may be the most toxic species, but as even stable PrP^Sc^ is proving difficult to determine the structure for, more temporary species are likely to be even harder to uncover.

Despite the evidence that argues against a direct toxic role of PrP^Sc^, a number of detrimental processes are associated with the misfolded protein. Among the best characterized are inhibition of the proteasome and over-activation of the UPR, but a number of other processes including synaptic disruptions, the initiation of apoptosis and induction of oxidative stress are also observed. A consensus on the molecular underpinnings of PrP^Sc^ associated toxicity is far from clear, and the heterogeneity of PrP^Sc^ may mean some of these processes are strain/structure dependent. It is also possible that the mere presence of an aggregating misfolded protein, rather than the intrinsic properties of PrP^Sc^, explain some of the associated toxicity. Similar damaging pathways are activated in other neurodegenerative diseases such as Alzheimer’s and Parkinson’s, where the identity of the misfolding proteins are different, but many similarities remain [88,160].

Unfortunately, treatment options for the prion diseases are extremely limited, but several strategies have been suggested. Reducing levels of PrP^C^ will remove the substrate of the conversion to PrP^Sc^ [161,162], but the increasing number of beneficial cellular roles attributed to PrP^C^ reduces the attractiveness of this approach. Another is to prevent the conversion of PrP^C^ to PrP^Sc^ by either stabilizing PrP^C^ or inhibiting the conversion process [163]. This has been the most popular approach, as it is amenable to small molecule or therapeutic antibody intervention, while preserving the function of PrP^C^ [164,165]. A third method is to accelerate clearance of PrP^Sc^ from the brain [166]. A new approach is to target the downstream effects of prion replication, by inhibiting processes such as the UPR. This is surprisingly effective, as the underlying prion conversion and aggregation is unaffected, but extensive neuroprotection is still observed [119,120,121]. The effectiveness of UPR inhibitors again illustrates the complicated relationship between PrP^Sc^ and neurotoxicity; the elucidation of these underlying molecular mechanisms will undoubtedly improve our ability to treat these devastating diseases.
